# Loneliness as a Risk Factor for Time to Care Home Entry for Older Adults Receiving Community Care

**DOI:** 10.1093/geroni/igaf010

**Published:** 2025-02-08

**Authors:** Sam Rickman, Jose-Luis Fernandez, Juliette Malley

**Affiliations:** Care Policy and Evaluation Centre (CPEC), London School of Economics, London, UK; Care Policy and Evaluation Centre (CPEC), London School of Economics, London, UK; Care Policy and Evaluation Centre (CPEC), London School of Economics, London, UK

**Keywords:** Adult social care, Day center, Large language models, Long-term care, Social inclusion

## Abstract

**Background and Objectives:**

International efforts to contain long-term care costs have prioritized personal care. However, reductions in services aimed at addressing loneliness or promoting social participation may affect demand for long-term care facilities. Research on the impact of loneliness on entry to residential or nursing care is based on survey data, which under-represents those with highest needs. Administrative records include such individuals and, unlike surveys, contain continuous data on service receipt, enabling accurate modeling of time to care home entry.

**Research Design and Methods:**

We use administrative data for 1 101 individuals receiving care in a London local authority. We extract loneliness from free text notes using a large language model and model its impact on care home entry 5 years after assessment, controlling for needs and demographics. We use logistic regression and a competing risks survival model to measure the time until care home entry.

**Results:**

The odds ratio for care home entry associated with loneliness is 1.45 with logistic regression (95% CI 1.04–2.01). The hazard ratio is 1.32 (95% CI 1.01–1.72) with a cause-specific model, and 1.39 (95% CI 1.08–1.79) using the Fine and Gray method. Among those most likely to enter a care home, the median time to entry is 9 months (95% CI 228–328 days) earlier for those who are lonely.

**Discussion and Implications:**

The hazard ratio of loneliness on care home entry is around the magnitude associated with gender, ethnicity, or living alone. However, loneliness is modifiable. Reductions to services for social participation, such as day centers, are likely to cause an increase in loneliness. We demonstrate that for those with the highest needs, loneliness is a significant risk factor for time until care home entry. Policymakers seeking to delay care home entry should consider the impact of services for loneliness.

**Translational significance:** This study models the time until entering a long-term care facility for older adults receiving publicly funded care, focusing on the impact of loneliness. The key finding is that loneliness significantly increases the risk of entry, accelerating it by a median of 9 months for those with the highest needs. Implications for translation include targeting services to reduce loneliness, particularly for those living alone or with cognitive impairments. This research suggests that policies should consider the impact of reduced social inclusion services on care home demand and provides a basis for predictive models using administrative data.

## Background and Objectives

Demand for long-term care (LTC) services is increasing internationally, and this trend is projected to continue (1–3). Although countries such as Japan and Norway are exceptions, the increased pressure on public funds has led to a “general tendency toward reconsidering and tightening the eligibility criteria for access to public LTC services” (4). For instance, Finland, Denmark, the United Kingdom, Sweden, and the Netherlands have recently experienced a retrenchment in community care services toward personal care and nursing tasks, with domestic and social support implicitly shifted to informal care networks [see (5–8)].

Although these policy changes aim to target resources toward those with the greatest needs, they may have unintended consequences. Services for promoting social participation such as day centers can lead to a reduction in loneliness (9), a factor associated with a higher risk of care home admission for older people observed in surveys (10). Yet there has been little analysis of the impact on care home entry of reducing the supply of such services to those with the highest needs, particularly for individuals receiving publicly funded care.

In this paper, we explore loneliness and social isolation as risk factors for care home entry among older publicly funded social care users, using English administrative data. In England, long-term care (generally called adult social care) provides support for people who need help with daily activities such as washing, dressing, and eating. Individuals requesting publicly funded care must undergo an assessment by their local authority under the Care Act 2014. Eligibility is based on a relatively high threshold of need, requiring individuals to be unable to independently complete 2 or more activities of daily living (ADLs) or instrumental activities of daily living (IADLs), with a significant impact on their well-being, alongside a means test. Around 400 000 people aged 65 and over in England receive publicly funded care, representing 3.6% of this age group, with the proportion rising to 11.7% among those aged 85 and over (11,12). The majority of community care users are estimated to be publicly funded (13). Care provision is predominantly related to ADLs, with services including personal care, shopping, meal preparation, housework, laundry, and accessing social or community activities.

We focus on moving to a residential or nursing care home, which is generally considered undesirable as it is associated with a loss of independence, dignity, and privacy and has high costs (14,15). In England, the total public expenditure on long-term care services in 2022/2023 was £15.1 billion, of which £6.6 billion funded care home places (16). Several studies have found that loneliness is associated with the risk of care home entry for older adults (10,17–21). If increased loneliness affects the risk of care home entry for publicly funded care users, this should be considered when calculating the effects of retrenchment of long-term care toward personal care.

However, most studies investigating the effect of loneliness use population survey data (20). Due to means-testing, retrenchment, and tightening eligibility criteria, statutory care users have a different needs profile to the general population, being more disabled, economically deprived, and reliant on formal care services. Such individuals are often under-represented in surveys due to systematic exclusions (22,23) and attrition (24–28). In the English case, there are marked differences in reported levels of need among older adults in the English Longitudinal Study of Ageing (ELSA) (29) who state they receive publicly funded care compared with administrative data. Although proportions of demographic information such as age and gender are similar, around half as many state they require support with personal care tasks (see Table 1). We cannot be confident that the findings from surveys that loneliness is a risk factor for care home entry for older people can be generalized to those with high levels of need—i.e., those at greatest risk of care home entry—who are under-represented in surveys.

**Table 1:** Comparison of Demographic and ADL Needs With ELSA

	Administrative data		ELSA			
			Weighted		Unweighted	
	*N* (%)	*N* Uniq	*N* (%)	*N* Uniq	%	95% CI
Awareness of risk (impaired)	806 (73%)	806	90 (21%)	81	20.5%	(16.1%, 24.9%)
Dressing (requires support)	877 (80%)	877	293 (69%)	233	68.9%	(63.5%, 74.3%)
Ethnicity (non-White)	364 (33%)	364	18 (4%)	15	4.2%	(1.8%, 6.6%)
Lives alone	608 (55%)	608	255 (60%)	203	61.2%	(55.1%, 67.3%)
Meals (requires support)	998 (91%)	998	276 (65%)	219	65.5%	(60.3%, 70.8%)
Memory (has needs)	664 (60%)	664	50 (12%)	49	24.8%	(18.0%, 31.7%)
Sex (F)	686 (62%)	686	267 (63%)	208	65.2%	(59.3%, 71.2%)
Shopping (requires support)	1066 (97%)	1066	336 (79%)	272	81.1%	(77.3%, 85.0%)
Toileting (requires support)	570 (52%)	570	160 (38%)	132	35.3%	(30.0%, 40.5%)
Unpaid care (receives)	819 (74%)	819	344 (81%)	277	82.8%	(78.7%, 86.9%)

*Notes*: ELSA: English Longitudinal Study of Ageing. Using pooled data from waves 6, 7, 8, and 9 of ELSA. *N* Uniq: total unique individuals across all waves. Weighted: using longitudinal weights provided with ELSA.

This paper examines whether loneliness or social isolation recorded at the time of a person’s initial assessment affects the time until an individual enters a care home, controlling for needs and demographic factors. Loneliness and social isolation are closely related but distinct concepts (30–32). Social isolation generally refers to an “objective lack of relationships,” whereas loneliness is a “subjective, distressing feeling” that arises when an individual’s desired quantity or quality of social connections is unmet (33). These concepts often overlap in their effects, as both loneliness and social isolation have been linked to adverse outcomes, including increased mortality among older adults (34). In this paper, we recognize the challenges in distinguishing loneliness from social isolation within free text social care records. Social workers may use terms like “lonely” or “isolated” interchangeably or imprecisely, describing either a subjective sense of loneliness or a more objective lack of social contact, or both. We adopt a pragmatic approach by considering loneliness and social isolation together, consistent with the integrated perspective seen in public health research (34–38). For the sake of brevity, except where explicitly distinguished from social isolation, we use the term “loneliness” in this paper to refer to our combined measure of loneliness or social isolation.

Our analysis differs from previous research in that we use administrative records. These records, collected by agencies in the course of service delivery, contain real-time information about service use for individuals receiving publicly funded care, allowing us to model time to care home entry. Administrative data has enabled researchers to establish that the probability of care home entry is associated with age, gender, disability, ethnicity, and depression [eg, (39)]. However, loneliness is not generally recorded as a structured indicator in administrative data and it has not been included in analyses using administrative records, or record-linkage models of socio-demographic variations in moves to care homes [eg, (40)].

Using records collated by agencies in the process of service delivery offers advantages. There is no attrition as health declines, so the records capture information on those with the highest need. These records contain continuous, time-variant service use data, allowing more precise estimation of the time until care home entry. Surveys provide snapshots at each wave. Researchers can impute the date of institutionalization between waves but this increases uncertainty (18,19).

It is essential to account for factors associated with both loneliness and care home entry to robustly examine their association. Care home entry is correlated with age, gender, ethnicity, functional impairment, and living alone (10,20,41,42), though this is moderated by receipt of unpaid care (19). Dementia, which has a bidirectional relationship with social participation (43,44), is a highly significant predictor of care home entry (17,19). Our analysis controls for age, sex, ethnicity, cognitive impairment, support required with ADL needs (personal care) and IADL needs (shopping and meal preparation), and living circumstances (receipt of unpaid care, living alone). Receipt of formal or unpaid care may affect the risk of care home entry (19). Survey-based research into the effect of loneliness on care home entry (19) has not controlled for care receipt. By including these covariates, we aim to isolate the effect of loneliness and ensure it is not conflated with other factors that influence the risk of care home entry. As a baseline model, we replicate the approach in (17), using logistic regression. To compare differences in the rate of care home entry over time between those identified as lonely or isolated and others, we use a survival model, accounting for competing risks, as not all (or even most) individuals will ever enter a care home (45).

We investigate three questions: First, does loneliness predict care home entry? Second, holding other factors equal, what is the difference in time to care home entry if an individual is lonely? Finally, does loneliness particularly increase the risk of care home entry in certain groups, such as women, those who are more physically disabled, or those with impaired cognition? This final question is important for understanding how best to target services to prevent care home entry.

## Research Design and Methods

### Data set

To explore the relationship between loneliness and care home entry, we use data from an inner London local authority. In England, under the Care Act 2014, every person seeking publicly funded care undergoes an assessment by a social care professional to establish their eligibility. This assessment generates a document recording information relevant to care needs such as functional ability to perform ADLs and IADLs, cognitive function, and unpaid care.

We received approval from the NHS Confidentiality Advisory Group (CAG) to use this data for this purpose and obtained ethical approval. A query was written to identify all individuals aged 65 or over on August 1, 2020 who had been receiving services arranged by the local authority for at least one year. The data set includes information for 3 046 individuals between January 1, 2015 and August 31, 2020. The export includes all needs assessment forms completed between January 2015 and August 2020. After an assessment is completed, if an individual receives care, services commissioned are recorded. The export includes individual-level, time-variant service use data with costs between January 2015 and August 2020.

Complexity in the data is that loneliness can be recorded at any time during an individual’s contact with care services. We resolve this by using loneliness at the time of initial needs assessment. There are 2 reasons for choosing this time point. First, we expect that needs to be comprehensively recorded at first contact. Second, if loneliness at first presentation to a local authority is a relevant factor for care home entry, it provides the greatest opportunity for intervention. There are 1 649 individuals in the exported data whose initial needs assessment occurs in the period of observation. Needs assessments were captured on different forms during the period. This was determined by policy changes and is not correlated with individual needs. After limiting the data set to forms that contained questions covering all relevant covariates, 1 331 individuals remained. We also exclude from the analysis individuals who enter care homes immediately after their first presentation to social care. These cases represent an event leading to a sudden development of care needs, such as a fall or stroke. There is no opportunity in such cases for local authorities to put in place preventative services for loneliness with a view to delaying care home entry. We limit the period of observation to 5 years from initial assessment. Our final data set contains 1 101 individuals. At the end of the period of observation, 252 people have entered a care home, 502 die before entering a care home and 347 are censored, that is, continue to receive care in the community at the end of the period of observation.

### Characteristics of Individuals in the Data

Information was captured using structured data fields and free text case notes. Structured fields are inherently machine-readable (46). In our data set, they record key demographic and personal information necessary for care planning and service delivery, such as age, gender, ethnicity, functional ability with ADLs and IADLs, and whether the individual lives alone. Free text fields can be included within needs assessment forms, or in distinct areas of case management systems to record information not covered elsewhere (“case notes”). In this study, we extract the loneliness measure from free text, and all needs-related covariates from structured data. We classified for loneliness or social isolation all free text notes recorded about the 1 101 individuals within 90 days of their initial assessment (*N* = 62 603). We present in Table 2 a breakdown of loneliness and care home entry by each covariate, including the p value for tests of independence, for categorical variables using a $\chi^{2}$ test, and for continuous variables Pr(>F) after an analysis of variance. We explore these relationships and contrast them with the regression output in the Discussion section.

**Table 2.** Descriptive Statistics

Variable	Levels	Care Home Entry				Loneliness or Isolation		
		Censored (%)	Care Home (%)	Death (%)	*p*	Not Lonely (%)	Lonely (%)	*p*
Lonely	No	258 (32.5)	152 (19.2)	383 (48.3)	<.001	793 (100)	0 (0)	
	Yes	89 (28.9)	100 (32.5)	119 (38.6)		0 (0)	308 (100)	
Sex	Female	235 (34.3)	149 (21.7)	302 (44.0)	.041	488 (71.1)	198 (28.9)	.438
	Male	112 (27.0)	103 (24.8)	200 (48.2)		305 (73.5)	110 (26.5)	
Personal care	Low/no needs	157 (35.4)	113 (25.5)	174 (39.2)	.005	301 (67.8)	143 (32.2)	.001
	Moderate	150 (30.5)	100 (20.4)	241 (49.1)		355 (72.3)	136 (27.7)	
	High	40 (24.1)	39 (23.5)	87 (52.4)		137 (82.5)	29 (17.5)	
Cognition	No/low needs	245 (35.3)	115 (16.6)	334 (48.1)	<.001	539 (77.7)	155 (22.3)	<.001
	Moderate	52 (26.8)	63 (32.5)	79 (40.7)		120 (61.9)	74 (38.1)	
	High	50 (23.5)	74 (34.7)	89 (41.8)		134 (62.9)	79 (37.1)	
Ethnicity	Non-white	128 (35.2)	72 (19.8)	164 (45.1)	.099	266 (73.1)	98 (26.9)	.635
	White	219 (29.7)	180 (24.4)	338 (45.9)		527 (71.5)	210 (28.5)	
Shopping/meals	Low/no needs	56 (39.4)	38 (26.8)	48 (33.8)	.003	96 (67.6)	46 (32.4)	.412
	Moderate	129 (33.2)	95 (24.5)	164 (42.3)		279 (71.9)	109 (28.1)	
	High	162 (28.4)	119 (20.8)	290 (50.8)		418 (73.2)	153 (26.8)	
Lives alone	No	162 (32.9)	94 (19.1)	237 (48.1)	.025	371 (75.3)	122 (24.7)	.037
	Yes	185 (30.4)	158 (26.0)	265 (43.6)		422 (69.4)	186 (30.6)	
Unpaid care	No	107 (37.9)	71 (25.2)	104 (36.9)	.002	207 (73.4)	75 (26.6)	.602
	Yes	240 (29.3)	181 (22.1)	398 (48.6)		586 (71.6)	233 (28.4)	
Has telecare	No	280 (33.3)	193 (22.9)	369 (43.8)	.053	618 (73.4)	224 (26.6)	.080
	Yes	67 (25.9)	59 (22.8)	133 (51.4)		175 (67.6)	84 (32.4)	
N Notes	Mean (SD)	513.4 (457.5)	691.6 (513.0)	522.9 (412.1)	<.001	531.7 (451.7)	627.4 (462.7)	.002
Age	Mean (SD)	83.2 (8.6)	85.9 (7.4)	84.4 (8.3)	<.001	84.0 (8.4)	85.4 (7.6)	.011
Cost DPs	Mean (SD)	9.1 (39.6)	2.6 (22.7)	9.7 (68.8)	.191	7.3 (45.2)	9.3 (68.2)	.575
Cost day care	Mean (SD)	5.9 (34.2)	8.1 (34.6)	2.8 (15.7)	.032	2.0 (21.5)	12.6 (38.0)	<.001
Cost homecare	Mean (SD)	118.5 (124.3)	125.1 (153.0)	125.6 (138.9)	.742	128.5 (142.7)	109.6 (123.6)	.041

*Notes*: DPs: direct payments. Day care represents services for social inclusion (day centers). N Notes is the total number of case notes. Costs are the cost of services put in place within 90 days of initial assessment.

### Model Parameters

Loneliness is extracted from free text as described in (47). The natural language processing model produces a binary classification for each sentence, indicating whether loneliness or social isolation is recorded. We consider an individual to be lonely or isolated at the time of assessment if they have at least one sentence in their needs assessment form and one sentence in case notes which is indicative of loneliness or social isolation. As information about social networks is extracted from free text records, which do not reliably distinguish between the related but distinct concepts of loneliness and social isolation, our indicator reflects individuals who are recorded as being either lonely or socially isolated.

As loneliness is based on free text, we also include in the model the number of case note sentences recorded about an individual (N Notes). This means any association between loneliness and care home entry cannot be explained by the natural language processing model being more likely to indicate loneliness where more case notes are recorded.

We also include in the model services received after the initial needs assessment, to control for the effect of service receipt and capture differences in need not reflected in service provision. We include the cost of home care, day care, and direct payments (DPs), as well as whether individuals receive telecare services. All covariates except loneliness are extracted from structured data. We limit the period of observation to 5 years after initial assessment.

### Models

We use logistic regression as a baseline model, and to compare results for statutory care users against the general older population in (17). However, logistic regression does not distinguish between an individual who enters a care home after one day and another who enters 2 years later, though this difference may be meaningful for those individuals. We also use a survival model with competing risks, to allow us to model the length of time that an individual spends outside a care home. The competing risks element of the model accounts for the fact that, unlike traditional survival models where the event of interest is death, not all individuals will enter a care home.

#### Logistic regression model

We use a logistic regression model, modeling care home entry as 1 (N=252), and not entering a care home as 0 (N=849), as specified in Equation 1.


$$\begin{array}{l} \begin{array}{ll} y & =\frac{e^{\beta_{0}+\beta_{1}lonely+\beta_{i}X_{i}}}{1+e^{\beta_{0}+\beta_{1}lonely+\beta_{i}X_{i}}}\;\;\;\;\;\;\;\;\;\;\;\;\;\;\;\;\;\;\;\;\;\;\;\;\; \end{array} \end{array}$$ (1)

where lonely is a binary variable indicating whether an individual was lonely at the time of initial assessment, and $X=\left(X_{2},X_{3},\ldots X_{i}\right)$, a vector of the explanatory variables set out in Table 2.

#### Survival model with competing risks

A survival model is a method to account for differences in time between the initial assessment and care home entry. However, care home entry (*N* = 252) is not the only possible outcome. It is also possible for individuals who are at high risk of care home entry to die prior to entering a care home (*N* = 502), as well as to be censored, that is, remain in the community at the end of the period of observation (*N* = 347). We therefore use a competing risks model. We use the Aalen–Johansen estimator, a generalization of the Kaplan–Meier approach (48) to estimate a cause-specific hazard function (49). Critics of the cause-specific estimator note that as individuals who die prior to the outcome of interest (in this case care home entry) are removed from the risk set, it can fail to capture the risk in a population despite accurately reflecting the sample, as it is not known in advance when individuals at risk will die (49). We therefore also estimate the subdistribution hazard using a Fine and Gray competing risks model, where the hazard function is defined as in (50). We fit 2 models to estimate the respective hazard ratios as specified in Equation 2.


$$\begin{array}{l} \begin{array}{l} h_{k,j}\left(t|lonely,X\right)=h_{0_{j,k}}\cdot e^{\beta_{1_{j,k}}}lonely\cdot e^{\beta_{i_{j,k}}X_{i}}\;\;\;\;\;\;\;\;\;\;\;\;\;\;\;\;\;\;\;\;\; \end{array} \end{array}$$ (2)

for k∈{1,2} and j∈{1,2}, where k:1=care home,2=deathj:1=cause-specific,2=subdistribution$X=\left(X_{2},X_{3},\ldots X_{i}\right)$, a vector of the explanatory variables set out in Table 2.

The advantage of the Fine and Gray approach is that it includes in the risk set individuals who enter another state (ie, we model the risk for individuals who die before time t, reflecting that in the population we do not know which individuals will die). Proponents of the cause-specific approach argue that the Fine and Gray approach can be difficult to interpret as it uses a risk set which does not exist (51). We subscribe to the view of Austin et al. (49) that cause-specific models are appropriate for interpreting individual covariates, and Fine and Gray is suitable for predicting the risk for individuals with different combinations of needs through the subdistribution hazard function. We present results for both models but prefer the cause-specific hazard ratios, and use the Fine and Gray model for generating predictions for sub-groups of individuals.

Both models multiply a base hazard rate by a vector of covariates, so assume proportional hazards. As this assumption was not satisfied for cognition or the number of sentences written at the time of the assessment (N notes), we stratified by these variables to avoid violating it. The number of sentences (n) is a continuous variable so to stratify we split it into low (n<440), medium (440≥n<1000), and high (n≥1000) to satisfy the assumption. In the Fine and Gray model, which requires reshaping the data into counting process format, the proportional hazards assumption was also not satisfied for home care costs at initial assessment, so again we stratified weekly cost (c) in £ into 3 groups, low (c<50), medium (50≥c<150) and high (c≥150). After stratification the proportional hazards assumption was satisfied for all variables in the model.

We also conducted additional analyses to interrogate the effect of loneliness on the oldest old, and the inclusion of living alone in the model:

1. Binary age specification: We specify age as a binary variable (<85 vs ≥85) by replacing the continuous and quadratic age terms in the main model.
2. Stratified age specification: We stratify our data set into the 2 age groups (<85 vs ≥85) and run the same models as in Equations 1 and 2 separately for each age group.

We include the results for the additional analyses in Supplementary Material. All analysis was undertaken with R 4.2.2 (52), using the survival package (45) for the competing risks models.

## Results

**Figure 1.**
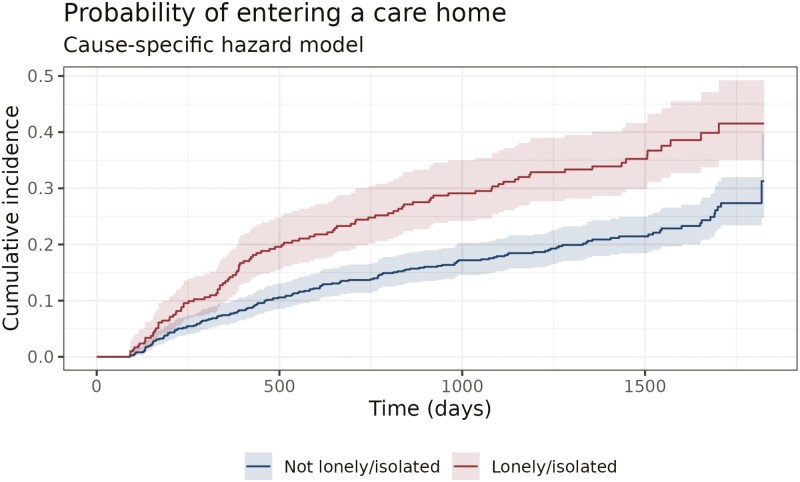
Cumulative incidence of care home entry based on loneliness status.

We present in Table 3 the output from the logistic regression model. Loneliness significantly (α=0.05) increases the risk of care home entry, with an odds ratio of 1.45 (95% CI 1.04–2.01). We present in Figure 1 the cumulative incidence of care home entry for individuals who are and are not identified as lonely or isolated at the time of initial assessment. The plot does not control for confounding factors, and we present the results of the regression, adjusting for covariates, in Table 4. The magnitude of the effect is similar in the competing risks models, with the presence of loneliness increasing either the odds ratio or the instantaneous risk of care home entry in the range of 1.32–1.39. Loneliness remains significant after accounting for other factors with which it is associated. In particular, the effect of loneliness cannot be explained by living alone, receipt of unpaid care, cognition, or functional ability, all of which were included in the model.

**Table 3.** Logistic Regression Model Output

Variable	Odds Ratio (CIs)	*p*	
*Loneliness*			
Lonely or Isolated	1.45 (1.04–2.01)	.027	^*^
*Demographics*			
Sex: male	1.27 (0.93–1.74)	.135	
Age	1.37 (0.96–1.98)	.087	^^^
Age^2	1.00 (1.00–1.00)	.134	
Ethnicity: White	1.41 (1.01–1.98)	.046	^*^
Lives alone	1.63 (1.16–2.31)	.005	^**^
Unpaid care	0.79 (0.55–1.14)	.203	
*Needs*			
N Notes	1.00 (1.00–1.00)	<.001	^***^
Personal care: moderate	0.76 (0.52–1.11)	.161	
Personal care: high	1.08 (0.62–1.89)	.783	
Cognition: moderate	2.76 (1.86–4.10)	<.001	^***^
Cognition: high	3.87 (2.57–5.86)	<.001	^***^
*Services*			
Shopping and meals: moderate	1.00 (0.62–1.64)	.989	
Shopping and meals: high	0.61 (0.35–1.06)	.075	^^^
Cost DPs	1.00 (0.99–1.00)	.173	
Cost day care	1.00 (1.00–1.01)	.321	
Cost homecare	1.00 (1.00–1.00)	.498	
Has telecare	0.92 (0.63–1.31)	.637	

*Note*: CIs = confidence intervals; DPs = direct payments.

^***^ *p* < .001; ^**^p < .01; ^*^p < .05; ^^^*p* < .1.

**Table 4.** Competing Risks Model Output (Hazard Ratios)

Variable	Cause-specific hazard (CIs)	*p*		Fine and Gray (CIs)	*p*	
*Loneliness*						
Lonely or isolated	1.32 (1.01–1.72)	.039	^*^	1.39 (1.08–1.79)	.009	^**^
*Demographics*						
Age	1.16 (0.84–1.60)	.369		1.27 (0.96–1.68)	.093	^
Age^^^2	1.00 (1.00–1.00)	.461		1.00 (1.00–1.00)	.136	
Ethnicity: White	1.42 (1.08–1.88)	.013	^*^	1.30 (1.00–1.69)	.047	^*^
Lives alone	1.54 (1.16–2.03)	.003	^**^	1.47 (1.14–1.91)	.003	^**^
Sex: male	1.34 (1.03–1.74)	.030	*	1.15 (0.90–1.46)	.278	
Unpaid care	0.92 (0.68–1.24)	.584		0.85 (0.65–1.12)	.248	
*Needs*						
Personal care: high	1.39 (0.85–2.29)	.189		1.08 (0.68–1.70)	.749	
Personal care: moderate	0.96 (0.69–1.32)	.786		0.81 (0.60–1.10)	.174	
Shopping and meals: high	0.65 (0.42–1.01)	.056	^^^	0.65 (0.43–0.99)	.043	^*^
Shopping and meals: moderate	0.88 (0.60–1.29)	.511		0.93 (0.65–1.33)	.683	
*Services*						
Cost DPs	1.00 (0.99–1.00)	.091	^^^	1.00 (0.99–1.00)	.119	
Cost day care	1.00 (1.00–1.00)	.784		1.00 (1.00–1.00)	.663	
Cost homecare	1.00 (1.00–1.00)					
Has telecare	0.82 (0.60–1.11)	.204		0.90 (0.68–1.19)	.457	

*Note*: CIs= confidence intervals; DPs = direct payments.

^***^ *p* < .001; ^**^p < .01; ^*^p < .05; ^^^*p* < .1.

The greatest predictor of care home entry in all models is cognition, which is consistent with the literature [eg (39)]. As we have stratified the survival models by cognition, a coefficient for cognition is not estimated in these models. Cognition does not meet the assumption because individuals with significant cognitive impairment at initial assessment are likely to enter a care home during the first year, unlike those individuals with no cognitive impairment. We compare the cumulative incidence in Supplementary Figure 1 in Supplementary Material. Although stratification means the model does not produce a coefficient, the way in which this assumption is violated highlights the paramount importance of cognition in determining time until care home entry, particularly in the first year after assessment.

The estimates from the alternatively specified models are consistent with the main results. The competing risks models which treat age as a binary variable find that being aged >85 is a significant predictor of care home entry, in contrast to age as a continuous variable in Table 4. The results for loneliness in this model are consistent with the main body of the paper, with very little change in the size of the coefficient or p values, indicating that loneliness is a robust predictor of care home entry regardless of how age is specified. We include the full results of these models in Supplementary Material.

We find similarly to Kersting (19) that using a model accounting for time to care home entry provides insight into the factors associated with care home entry. Gender is significant in the cause-specific hazard model, though not in the logistic regression. We see in Table 2 that 22% of women in the sample ultimately enter care homes, compared with 25% of men, and the $\chi^{2}$ test indicates this difference is significant. However, Table 3 shows that controlling for other covariates, in a logistic regression the difference disappears. Men generally tend to die earlier, and in our data are at risk of care home entry for 603 days, whereas women are at risk for 682 days. A one-sided t test indicates this is also a significant difference (p<0.001). This difference means the yearly rate of care home entry for men is about 20% higher than for women, impacting the significance of the cause-specific survival model. Conversely, in the Fine and Gray model, gender is not significant. This is because individuals who have died remain in the risk set for care home entry. As men also die earlier and more often than women, this increases the risk set for males entering care homes more than it does for women, so the overall difference in rate is diluted. An advantage of the Fine and Gray model is that it does not assume knowledge of the future, such as knowing who will die (50). However, it is well-established that mortality rates for men are higher. Although this remains the case, the cause-specific hazard ratio is a more appropriate measure, as it accounts for the fact that men on average spend less time at risk of care home entry. There is otherwise little difference between the output of the 2 competing risk models.

We similarly see that a survival model gives some insight into groups who may be more likely to enter care homes quickly. IADLs (high support needs with shopping and meals) appear to be significant using Fine and Gray and not in the cause-specific hazard model, but this is primarily a reflection of the decision to set α=0.05, as the p values are both very close to this, at 0.056 and 0.043, respectively. The hazard ratio is <1, indicating individuals who live alone, are lonely, have a significant cognitive impairment, and are independent with shopping and meal preparation are at the highest risk of care home entry. We hypothesize that such individuals may be felt to be at particular risk, for example of wandering, and that a model which accounts for time to care home entry can distinguish that such individuals enter care homes particularly quickly. However, this small group is on the boundary of significance in both and is not a focus of this research. More research would be required to definitively identify this phenomenon, ideally with a larger sample to allow for the introduction of interaction effects.

**Figure 2.**
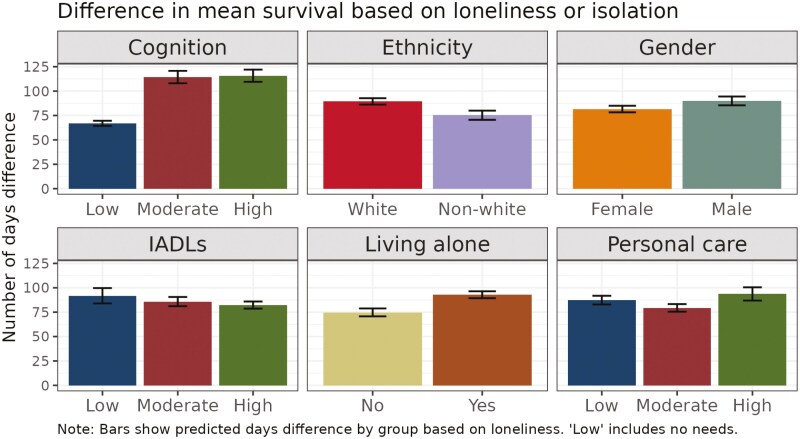
Difference in mean time until care home entry by group.

To measure the effect size of loneliness, we created 2 synthetic data sets, both based on our original data and identical in number of individuals and all characteristics, except in one data set all records were marked as lonely, and in the other none were. We generated survival curves for restricted mean survival times (RMST), representing the average time to care home entry up to the specified time horizon, using the subdistribution hazard from the Fine and Gray model. We present in Figure 2 the mean difference in RMST by group. Although loneliness increases the risk of care home entry for all individuals, the difference varies considerably between groups. In particular, lonely individuals with a cognitive impairment enter care homes a mean of 115 days earlier than those with a cognitive impairment who are not lonely. Conversely, in those without a cognitive impairment, the difference is 67 days. Loneliness makes a difference of around 3 months across all levels of personal care, IADLs (shopping and meal preparation), and for both men and women. The overall mean difference in RMST across all individuals is also around 3 months, 85 days (95% CI 82.05–87.47 days). We also see significant differences in the impact of loneliness on time to care home entry based on ethnicity and living alone. As a sensitivity check, we also examined the median differences in RMST across individuals. The results aligned closely with those based on mean RMST, showing that lonely individuals with *High* levels of cognitive impairment experience greater differences in time to care home entry if they are lonely (109 days) than those with *Low/no needs* (60 days), and similar patterns were observed for other variables, such as ethnicity and living alone. These findings confirm the robustness of the observed trends across different measures of central tendency.

However, RMST includes the very long survival times seen in most people, who never enter a care home. For many combinations of covariates, at the end of the observation half of individuals will not have entered a care home, so it is not possible to present the median survival time, that is, the time at which the probability of having entered a care home is 0.5. However, where we can compare, we can expect median time to care home entry considerably earlier among those who are lonely or socially isolated than those with equivalent needs who are not, with an overall difference of 278 days, or around 9 months (95% CI 228.03–328.46 days). This difference is larger than RMST which, while informative about the average effect across the entire sample, is influenced by the inclusion of individuals who do not enter care homes during the observation period, and thus may not fully capture the more pronounced effects of loneliness observed in those most at risk. Although there are differences between RMST and the median time until care home entry, as they describe different populations, both indicate that those who are lonely can be expected to enter a care home several months earlier than their counterparts.

### Limitations

Although loneliness is a significant predictor, we cannot rule out that it is correlated with an unobserved variable that increases the risk of care home entry, such as personality. If this were the case, then it may be that reducing loneliness without affecting the unmeasured variable would not alter the risk of care home entry. Furthermore, there are some health-related risk factors that are known to be significant but not included in our data set, such as physical illness (though we proxy this through ADL needs) and hospitalization (39). We also do not include wealth, which is a significant predictor of care home entry in the general population (17). However, all individuals receiving publicly funded care must have income and capital below nationally set thresholds, so this is unlikely to be an important omission.

A strength of our data set is that it is a large enough sample of publicly funded care users to find significant results. However, a limitation is that all data used in this analysis is obtained from one source, a local authority case management system. We think it is likely that the results generalize at least to similar areas, but the data does not allow us to empirically test this. Additionally, as there were only 252 individuals who entered a care home, we are uncertain about the interpretation of the apparent lack of significance of some results. For example, the magnitude of high personal care ADL needs is greater than 1, but the p values are large. We do not know whether, if more data were gathered, we would see a significant effect.

Similarly, we would have liked to investigate the interaction of loneliness with service use, and with needs-related and demographic characteristics, but were unable to do so with a data set of this size. We would be more confident about generalizability and interactions if we had more data. However, as this is the entire cohort from a local authority, we would need to include other geographical areas. Such an analysis would be considerably more complex. Administrative records in England are collected by 152 local authorities, each using a variety of forms and processes. Combining data across authorities presents practical challenges, as does interpreting results. Such a project may be feasible but would require considerably more investment in collecting and cleaning data as well as more complex theoretical and statistical models.

Another limitation is, as set out in our Methods section, our indicator of loneliness is whether a worker has recorded that an individual is lonely, which is a proxy for true loneliness. However, administrative data is not recorded for research purposes and its accuracy can depend on the incentives of those creating the data, which may lead to nonrandom bias (53,54). There can be an under-identification of needs in administrative records (55–57) which could lead to residual confounding. For example, although our analysis controls for cognitive impairment, if the measure of cognitive impairment in our data set does not fully capture cognitive ability, some aspects of the relationship between cognition may still affect the results. Furthermore, the interplay between loneliness and cognition, including the bidirectional relationship between these factors, may also influence pathways to care home entry in ways that cannot be discerned from our data and warrant further exploration. However, this issue is not unique to administrative records, which record needs more accurately than surveys (58) and have the advantage of capturing a wider range of individuals, particularly those with the highest needs. Although no data set is without limitations, our data includes individual-level, needs-related information linked to time-variant service use data. By controlling for known predictors of care home entry, including cognitive impairment, functional abilities, and demographic factors, we aim to minimize residual confounding and ensure a more robust analysis of the relationship between loneliness and care home entry.

Additionally, as our period of observation was until August 2020, there may have been some impact of the pandemic on our results. However, although there was an overlap with the COVID-19 pandemic, it is likely its impact on our analysis is minimal. The data set includes only individuals who have been receiving long-term care services for at least one year. By August 2020, the end of the period of observation, this means that individuals would have started receiving services in August 2019, 6 months before the beginning of the pandemic. During the period March–August 2020, 15 individuals entered care homes, compared with 14 in the same period the previous year, suggesting that the pandemic was not a significant factor influencing care home entry in this data set.

A final limitation is that our data does not allow us to distinguish loneliness from social isolation and that although both constructs are continuous, our measure is binary. Furthermore, neither loneliness nor social isolation is one-dimensional. Although social isolation is typically seen as an objective measure (33), it can be assessed through the frequency, quality, or type of contact (59). Similarly, loneliness can be divided into emotional and social dimensions (60). We would prefer to have been able to disambiguate these concepts, as there are individuals who are lonely and not socially isolated, and vice versa, but our binary, combined measure of loneliness and social isolation does not capture these distinctions. Researchers using clinical psychiatric notes have developed a free text metric of loneliness that distinguishes between emotional loneliness and a lack of social networks (61). We could not find a way to derive a measure of the intensity or type of loneliness or isolation from the administrative data we had access to, but this would be a valuable direction for future research, particularly as large language models continue to develop. However, the interventions commissioned as part of a long-term care package are likely to include day centers, support to access activities in the community, or befriending. These interventions may be appropriate for loneliness or social isolation, so we do not think that this limitation detracts from the conclusion that more research is needed into the effectiveness of such interventions on care home entry.

## Discussion and Policy Implications

In this study, we investigate whether loneliness predicts care home entry for publicly funded care users. We find loneliness is a significant predictor of care home entry, controlling for other factors. The hazard ratio of loneliness is around 1.32–1.39 using a survival model. This is consistent with other studies which find the effect of loneliness on care home entry is less than that of impaired cognition, but around the same magnitude as the effect of ethnicity, living alone, and gender (10). However, loneliness is modifiable. Our second research question was to determine the magnitude of the effect of loneliness. We find that, holding other factors constant, the difference in median time to care home entry if an individual is lonely is around 9 months in those groups where this could be compared, and the mean difference across all individuals is around 5 months. Finally, we sought to establish whether loneliness particularly increases the risk of care home entry in certain groups. We find that although loneliness increases the risk of care home entry generally, it particularly does so for those individuals with a cognitive impairment.

Our analysis underscores the importance of research into those with the highest needs, such as publicly funded care users, who are demographically different even to the older adults in survey data who report they receive publicly funded care. We see this in the effect of age, which (at α=0.05) is not significant as a continuous variable in the regression results, but has been found to be a predictor of care home entry for the general population of older adults [eg (40,62)]. We note that although the incremental effect of age is not significant, there is an effect of being over 85. We discuss this in Supplementary Material. This highlights the importance of research into individuals with care needs, where the same factors cannot necessarily be used to distinguish individual care trajectories from the general population. We attribute these differences to the fact that the individuals in our data are simply a different group to those within survey data, with less variance in health, wealth, and age.

The finding that loneliness has the largest impact on people with a cognitive impairment suggests services that aim to delay care home entry should be targeted particularly towards this group. This raises questions about equity. If there are 2 individuals who are both lonely, is it reasonable to provide only one person with services promoting social participation, based on potential future savings to public funds? This is an ethical question, which is beyond the scope of this paper. Future research which addresses some of the limitations we raise, about quantifying the degree or type of loneliness or social isolation, may support practical approaches to such questions.

Our research indicates that care commissioners should consider the effect of care home entry in their determinations about funding services to reduce loneliness, which tend to be much less costly than residential or nursing care homes [see eg (63)]. Social interventions, such as day centers, befriending schemes, or group activities often target both loneliness and social isolation (64). The distinction between loneliness and social isolation may be more salient for commissioners of health services, as psychological interventions tend to target loneliness specifically (although group-based psychological activities may also improve social networks) (64). Policymakers should be aware that interventions for loneliness do not necessarily address social isolation and vice versa when commissioning services. However, evidence for the pathways through which loneliness and isolation contribute to care home entry, and the impact of interventions remains inconclusive, partly due to the heterogeneity of designs and limited scalability of successful programs (43,65). Day center services can reduce loneliness, with volunteer-led services particularly effective (9). As lonely older adults enter care homes sooner, it seems plausible that interventions that reduce loneliness would delay care home admission. However, our research cannot conclude this. Loneliness has physiological effects (66). A lonely individual may have experienced an accumulation of such effects, leading to an increased risk of care home entry by the time of their first assessment by long-term care services. On the other hand, care home entry risk may be determined by the contemporaneous physical or psychosocial effects of loneliness, which can be ameliorated by intervention. Future research could use experimental (or quasi-experimental) methods to establish the impact of day care or other interventions for people experiencing loneliness on care home entry.

Overall, our paper is important because there have been many changes to the remit of long-term care services, on the legitimate basis of cost-containment. However, it is possible that there are substitution effects, with a reduction in services that address loneliness increasing demand for residential or nursing care. Our paper demonstrates that for those with the highest care needs, loneliness is a risk factor for care home entry, with a median time until care home 9 months earlier for those who are lonely. Targeting services to those with the highest need is essential. Universal preventative services for loneliness are unlikely to be cost-saving (67), as it is inefficient to provide relatively expensive services to many individuals who are unlikely to enter a care home. This paper indicates that those at the highest risk of care home entry are those who are lonely, live alone, are over 85, and have a cognitive impairment. We also describe how individuals in administrative data can have higher needs than those in survey data who report they receive statutory care. This means the factors that determine care home entry for individuals with the highest needs—such as publicly funded care users—are not necessarily the same as the factors for older adults in surveys. Commissioners and policymakers require such information to target services.

An advantage of a model based on administrative data is that it could be developed into a product that can be integrated into case management systems to produce real-time predictions of risk of care home entry. The free text model could establish whether a worker has recorded loneliness. Furthermore, the number of case note sentences written in the first 90 days is itself a significant predictor of time to care home entry in the next 5 years. We hypothesize that this might be because the volume of notes captures a measure of complexity of the case that is not a function of care needs. Based on the results of this paper, local authorities could automatically generate the risk of care home entry for an individual over the next 5 years based on their case management records 90 days after the initial assessment. This would allow them to identify those at greatest risk of care home entry and target services accordingly. The adoption of technological innovation in care depends not just on its utility, but also on organizational and implementation factors (68), and further work would be required to develop such a product in a way that it would be trusted and adopted.

This study has found a statistically significant and meaningful effect of loneliness on the risk of care home entry. Our work builds on previous research, such as Hanratty et al. (17). We show the importance of research using administrative records, as survey data may not capture those with the highest needs. We demonstrate that among users of statutory care services in a London local authority, lonely older adults enter care homes sooner. It seems plausible that interventions that reduce loneliness may delay care home admission. At the moment, it is not possible to definitively state this or quantify the magnitude of such an effect. This means policymakers and care commissioners are unable to accurately ascertain the impact of retrenchment of long-term care away from such services. More research is required to determine the effectiveness of interventions for loneliness on time until care home entry.

## Supplementary Material

igaf010_suppl_Supplementary_Material

## Data Availability

The data that support the findings of this study are individual-level, administrative care records. This is identifiable human data and restrictions apply to the availability of these data, which were used under licence for the current study, and so are not publicly available. It is not possible to share this data publicly as individual privacy could be compromised. The study was not preregistered. All methods and analyses were conducted in accordance with the study’s aims and objectives, as outlined in the manuscript.
