# Supplementary material for: Loneliness as a Risk Factor for Time to Care Home Entry for Older Adults Receiving Community Care
Source: Innov Aging. 2025 Feb 8;9(6):igaf010. doi: 10.1093/geroni/igaf010 (PMC12149530; doi:10.1093/geroni/igaf010)
Supplement: igaf010_suppl_Supplementary_Material [file igaf010_suppl_supplementary_material.docx]

# ***Innovation in Aging* Supplementary Material: Rickman, Fernandez, & Malley. Loneliness as a risk factor for time to care home entry for older adults receiving community care.**

## **Section 1: Cumulative incidence stratified by cognition**

We show in Supplementary [Figure](#fig-cog-preds)1 the predicted cumulative incidence of care home entry for those with and without a cognitive impairment, holding all continuous variables at their mean, the categorical variables to the middle value, and the binary variables to 0 (i.e. these curves are for a woman who lives alone). These demonstrate the impact of cognition on results, and why the proportional hazards assumption is not met for cognition.


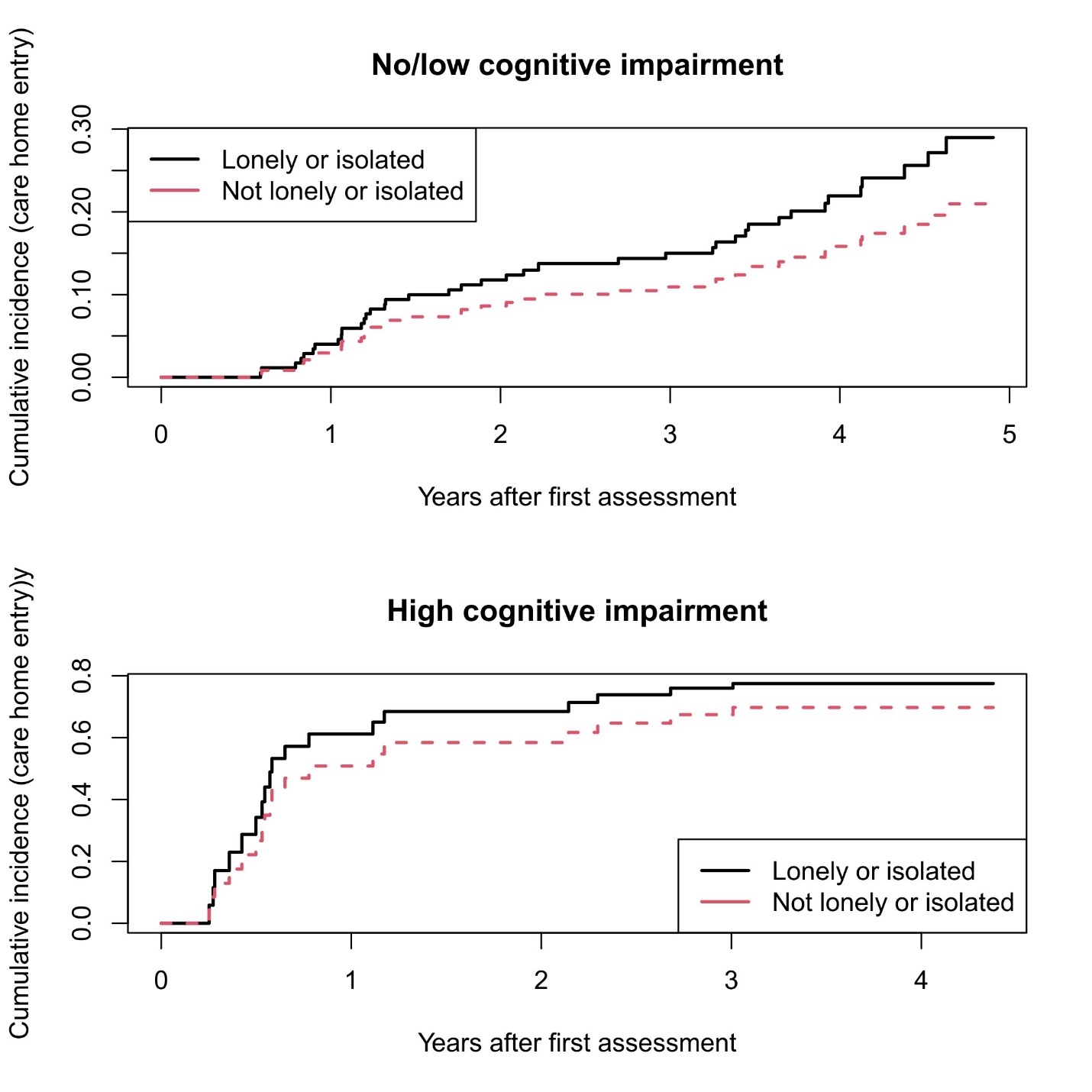


Supplementary Figure 1: Predicted cumulative incidence of care home entry stratified by cognitive impairment

## **Section 2: Exploring the role of age in loneliness and care home entry**

To better understand the relationship between age, loneliness, and care home entry, we conducted two supplementary analyses. First, we re-specified age as a binary variable, aged $<85$ ($N=570$) and $\geq85$ ($N=531$), to assess whether this approach influences the results. Second, we stratified our dataset into two age groups to examine how the effect of loneliness differs across these groups. The conclusion of these analyses is that the oldest older adults are at higher risk of care home entry in five years. Loneliness remains an important predictor of care home entry in these models. We present the output in full below.

### Impact of binary age specification on loneliness and care home entry

As a further analysis of the impact of age on our results, we fitted a model where age is specified as binary rather than continuous. We split individuals into aged $<85$ ($N=570$) and $\geq85$ ($N=531$), removing the quadratic age term ($\text{age}^{2}$) from the model specification. For the Fine & Gray model, this led to the violation of the proportional hazards assumption for the cost of day care services, so we stratified by individuals based on whether they received day care or not. After this step, the assumption was satisified. We present in Supplementary [Table 1](#tbl-logregagebinary) the output from the logistic regression model and in Supplementary [Table 2](#tbl-competingrisksagebinary) the output from the competing risks model.

The results for loneliness are similar to those in the main body of the paper. In the logistic regression and cause-specific hazard models, magnitude of the loneliness coefficient is slightly greater than the originally specified models, at 1.47 compared with 1.45 and 1.33 compared with 1.32, respectively, and the the $p$ values are slightly smaller. In the Fine & Gray model, we see the reverse, with the coefficient slightly smaller (1.36 compared with 1.39) and the $p$ value slightly larger but still significant at $a=0.05$. The inclusion of age as a binary variable does not meaningfully change the results for loneliness in any of the models. However, it is of note that age is now significant ($p<0.05$) in all three models, with a coefficient of 1.88 in the logistic regression model, 1.61 in the cause-specific hazard model and 1.42 in the Fine & Gray model. These results suggest that individuals aged 85 and older have an increased likelihood of care home entry compared to those under 85, which is consistent with the literature [e.g. 41]. The 95% confidence intervals for the estimates of all three models overlap, so it may not be appropriate to place too much interpretation on the differences between the models. These findings underscore that, while age is an important factor, loneliness remains a robust predictor of care home entry, regardless of how age is specified.

*Supplementary Table 1: Logistic regression model output (binary age specification)*

|  | Odds ratio |  |
| --- | --- | --- |
| Lonely or Isolated | 1.47 (1.06-2.04, p=0.021) | * |
| Sex: Male | 1.25 (0.91-1.71, p=0.158) |  |
| Age85+ | 1.88 (1.38-2.57, p<0.001) | *** |
| Ethnicity: White | 1.36 (0.98-1.91, p=0.071) | . |
| Lives Alone | 1.64 (1.16-2.32, p=0.005) | ** |
| Unpaid Care | 0.83 (0.58-1.19, p=0.310) |  |
| N Notes | 1.00 (1.00-1.00, p<0.001) | *** |
| Personal care: Moderate | 0.79 (0.54-1.14, p=0.209) |  |
| Personal care: High | 1.10 (0.63-1.93, p=0.733) |  |
| Cognition: Moderate | 2.78 (1.87-4.12, p<0.001) | *** |
| Cognition: High | 3.83 (2.54-5.80, p<0.001) | *** |
| Shopping and Meals: Moderate | 1.02 (0.63-1.66, p=0.938) |  |
| Shopping and Meals: High | 0.63 (0.37-1.09, p=0.095) | . |
| Cost DPs | 1.00 (0.99-1.00, p=0.152) |  |
| Cost Daycare | 1.00 (1.00-1.01, p=0.345) |  |
| Cost Homecare | 1.00 (1.00-1.00, p=0.502) |  |
| Has Telecare | 0.94 (0.65-1.35, p=0.756) |  |

**** p < 0.001; ** p <0.01; * p <0.05; p <0.1*

*Supplementary Table 2: Competing risks model output (binary age specification)*

|  | Cause-specific hazard |  | Fine & Gray |  |
| --- | --- | --- | --- | --- |
| Lonely or Isolated | 1.33 (1.02-1.73, p=0.036) | * | 1.36 (1.05-1.76, p=0.020) | * |
| Age85+ | 1.61 (1.24-2.08, p<0.001) | *** | 1.42 (1.12-1.79, p=0.004) | ** |
| Ethnicity: White | 1.37 (1.04-1.81, p=0.026) | * | 1.25 (0.96-1.62, p=0.095) | . |
| Lives Alone | 1.55 (1.18-2.05, p=0.002) | ** | 1.47 (1.12-1.91, p=0.005) | ** |
| Sex: Male | 1.29 (0.99-1.67, p=0.058) | . | 1.13 (0.88-1.44, p=0.340) |  |
| Unpaid Care | 0.95 (0.70-1.27, p=0.713) |  | 0.92 (0.70-1.21, p=0.555) |  |
| Personal care: High | 1.43 (0.87-2.34, p=0.159) |  | 1.06 (0.67-1.67, p=0.806) |  |
| Personal care: Moderate | 0.97 (0.71-1.34, p=0.875) |  | 0.78 (0.57-1.06, p=0.115) |  |
| Shopping and Meals: High | 0.67 (0.43-1.03, p=0.070) | . | 0.63 (0.41-0.95, p=0.029) | * |
| Shopping and Meals: Moderate | 0.89 (0.60-1.31, p=0.547) |  | 0.91 (0.63-1.30, p=0.592) |  |
| Cost DPs | 1.00 (0.99-1.00, p=0.079) | . | 1.00 (0.99-1.00, p=0.119) |  |
| Cost Daycare | 1.00 (1.00-1.00, p=0.769) |  |  |  |
| Cost Homecare | 1.00 (1.00-1.00, p=0.770) |  |  |  |
| Has Telecare | 0.82 (0.60-1.12, p=0.207) |  | 0.93 (0.71-1.23, p=0.629) |  |

**** p < 0.001; ** p <0.01; * p <0.05; p <0.1*

### **Age-stratified analysis of loneliness and care home entry**

To further explore the influence of age, we stratified our dataset into these two groups, aged $<85$ ($N=570$) and $\geq85$ ($N=531$), and ran the same model as in Equations 1 and 2 separately for each age group. We include the results for the logistic regression in Supplementary [Table](#tbl-logregagesplit)3 and for the competing risks model in Supplementary [Table](#tbl-crmodel85plus)4 The pattern in all three cases is that the coefficient for loneliness for those aged $\geq85$ is statistically significant at $\alpha=0.05$ and is in the range of 1.59 - 1.78 (compared with around 1.3 - 1.4 in the main models).

The coefficients for loneliness for older adults aged under 85 are 1.14 - 1.22 across the models and no longer statistically significant. This suggests the effect of loneliness on care home entry for younger adults is not as strong. This is in part because the overall risk of care home entry is lower for younger older adults. Combined with splitting the data in half, this may have reduced the power to detect a significant effect. These factors suggest that longer follow-up periods may be necessary to fully understand the impact of loneliness on care home entry among younger older adults, and that loneliness is an important predictor of care home entry in the oldest older adults.

*Supplementary Table 3: Logistic regression model output (stratified age specification)*

|  | Age <85 |  | Age 85+ |  |
| --- | --- | --- | --- | --- |
|  | Odds ratio |  | Odds ratio |  |
| Lonely or Isolated | 1.15 (0.65-1.99, p=0.622) |  | 1.68 (1.11-2.55, p=0.015) | * |
| Age | 1.05 (1.00-1.11, p=0.044) | * | 1.00 (0.95-1.05, p=0.982) |  |
| Ethnicity: White | 1.90 (1.12-3.32, p=0.021) | * | 1.12 (0.73-1.75, p=0.604) |  |
| Lives Alone | 1.29 (0.74-2.28, p=0.375) |  | 2.01 (1.29-3.17, p=0.002) | ** |
| Sex: Male | 0.97 (0.59-1.59, p=0.906) |  | 1.59 (1.04-2.41, p=0.030) | * |
| Unpaid Care | 0.57 (0.33-1.02, p=0.056) | . | 1.02 (0.63-1.67, p=0.927) |  |
| Cognition: High | 5.27 (2.66-10.55, p<0.001) | *** | 3.09 (1.83-5.25, p<0.001) | *** |
| Cognition: Moderate | 3.35 (1.75-6.39, p<0.001) | *** | 2.58 (1.54-4.30, p<0.001) | *** |
| N Notes | 1.00 (1.00-1.00, p<0.001) | *** | 1.00 (1.00-1.00, p=0.013) | * |
| Personal care: High | 1.09 (0.44-2.64, p=0.857) |  | 1.14 (0.54-2.38, p=0.727) |  |
| Personal care: Moderate | 0.67 (0.35-1.27, p=0.220) |  | 0.81 (0.50-1.31, p=0.394) |  |
| Shopping and Meals: High | 0.61 (0.25-1.47, p=0.264) |  | 0.60 (0.30-1.23, p=0.160) |  |
| Shopping and Meals: Moderate | 0.90 (0.42-1.97, p=0.793) |  | 1.03 (0.54-1.97, p=0.937) |  |
| Cost DPs | 1.00 (0.98-1.00, p=0.319) |  | 1.00 (0.99-1.00, p=0.427) |  |
| Cost Daycare | 1.01 (1.00-1.01, p=0.100) | . | 1.00 (0.99-1.01, p=0.845) |  |
| Cost Homecare | 1.00 (1.00-1.00, p=0.552) |  | 1.00 (1.00-1.00, p=0.548) |  |
| Has Telecare | 1.06 (0.56-1.95, p=0.860) |  | 0.83 (0.52-1.31, p=0.427) |  |

**** p < 0.001; ** p <0.01; * p <0.05; p <0.1*

*Supplementary Table 4: Competing risks model output (stratified age specification)*

|  | Age <85 | | |  | Age 85+ | | |
| --- | --- | --- | --- | --- | --- | --- | --- |
|  | Cause-specific |  | Fine & Gray |  | Cause-specific |  | Fine & Gray |
|  | Hazard ratio |  | Hazard ratio |  | Hazard ratio |  | Hazard ratio |
| Lonely or Isolated | 1.22 (0.76-1.96, p=0.404) |  | 1.14 (0.74-1.78, p=0.550) |  | 1.78 (1.24-2.56, p=0.002) | ** | 1.59 (1.16-2.18, p=0.004) |
| Ethnicity: White | 1.97 (1.19-3.27, p=0.009) | ** | 1.62 (1.07-2.44, p=0.022) | * | 1.01 (0.66-1.56, p=0.948) |  | 1.09 (0.77-1.54, p=0.624) |
| Lives Alone | 1.58 (0.98-2.56, p=0.063) | . | 1.35 (0.86-2.10, p=0.188) |  | 1.84 (1.18-2.86, p=0.007) | ** | 1.84 (1.26-2.69, p=0.002) |
| Unpaid Care | 0.74 (0.43-1.28, p=0.286) |  | 0.67 (0.42-1.06, p=0.087) | . | 1.06 (0.69-1.62, p=0.803) |  | 1.17 (0.78-1.74, p=0.451) |
| Personal care: High | 1.11 (0.47-2.63, p=0.817) |  | 1.06 (0.49-2.31, p=0.884) |  | 1.40 (0.68-2.89, p=0.364) |  | 1.17 (0.65-2.10, p=0.600) |
| Personal care: Moderate | 1.25 (0.75-2.10, p=0.392) |  | 0.81 (0.47-1.39, p=0.439) |  | 0.87 (0.55-1.36, p=0.535) |  | 0.87 (0.58-1.28, p=0.477) |
| Shopping and Meals: High | 0.63 (0.29-1.36, p=0.238) |  | 0.65 (0.35-1.20, p=0.169) |  | 0.85 (0.48-1.53, p=0.596) |  | 0.72 (0.41-1.27, p=0.258) |
| Shopping and Meals: Moderate | 0.80 (0.37-1.72, p=0.571) |  | 0.86 (0.48-1.52, p=0.598) |  | 0.93 (0.52-1.66, p=0.796) |  | 1.06 (0.67-1.69, p=0.791) |
| Cost DPs | 1.00 (0.99-1.00, p=0.226) |  | 1.00 (0.99-1.00, p=0.138) |  | 1.00 (0.99-1.01, p=0.569) |  | 1.00 (0.99-1.00, p=0.388) |
| Cost Daycare | 1.00 (0.99-1.01, p=0.822) |  | 1.00 (1.00-1.01, p=0.535) |  | 1.00 (0.99-1.00, p=0.689) |  | 1.00 (1.00-1.00, p=0.912) |
| Has Telecare | 0.93 (0.55-1.59, p=0.799) |  | 0.88 (0.54-1.45, p=0.618) |  | 0.83 (0.56-1.25, p=0.385) |  | 0.86 (0.61-1.23, p=0.424) |

**** p < 0.001; ** p <0.01; * p <0.05; p <0.1*
